# Effects of Aging on Cortical Neural Dynamics and Local Sleep Homeostasis in Mice

**DOI:** 10.1523/JNEUROSCI.2513-17.2018

**Published:** 2018-04-18

**Authors:** Laura E. McKillop, Simon P. Fisher, Nanyi Cui, Stuart N. Peirson, Russell G. Foster, Keith A. Wafford, Vladyslav V. Vyazovskiy

**Affiliations:** ^1^Department of Physiology, Anatomy and Genetics, University of Oxford, Oxford OX1 3PT, UK,; ^2^Sleep and Circadian Neuroscience Institute, Oxford Molecular Pathology Institute, Sir William Dunn School of Pathology, Oxford OX1 3RE, UK, and; ^3^Eli Lilly & Company Ltd, Erl Wood Manor, Windlesham, Surrey GU20 6PH, UK

**Keywords:** aging, mice, neocortex, sleep

## Abstract

Healthy aging is associated with marked effects on sleep, including its daily amount and architecture, as well as the specific EEG oscillations. Neither the neurophysiological underpinnings nor the biological significance of these changes are understood, and crucially the question remains whether aging is associated with reduced sleep need or a diminished capacity to generate sufficient sleep. Here we tested the hypothesis that aging may affect local cortical networks, disrupting the capacity to generate and sustain sleep oscillations, and with it the local homeostatic response to sleep loss. We performed chronic recordings of cortical neural activity and local field potentials from the motor cortex in young and older male C57BL/6J mice, during spontaneous waking and sleep, as well as during sleep after sleep deprivation. In older animals, we observed an increase in the incidence of non-rapid eye movement sleep local field potential slow waves and their associated neuronal silent (OFF) periods, whereas the overall pattern of state-dependent cortical neuronal firing was generally similar between ages. Furthermore, we observed that the response to sleep deprivation at the level of local cortical network activity was not affected by aging. Our data thus suggest that the local cortical neural dynamics and local sleep homeostatic mechanisms, at least in the motor cortex, are not impaired during healthy senescence in mice. This indicates that powerful protective or compensatory mechanisms may exist to maintain neuronal function stable across the life span, counteracting global changes in sleep amount and architecture.

**SIGNIFICANCE STATEMENT** The biological significance of age-dependent changes in sleep is unknown but may reflect either a diminished sleep need or a reduced capacity to generate deep sleep stages. As aging has been linked to profound disruptions in cortical sleep oscillations and because sleep need is reflected in specific patterns of cortical activity, we performed chronic electrophysiological recordings of cortical neural activity during waking, sleep, and after sleep deprivation from young and older mice. We found that all main hallmarks of cortical activity during spontaneous sleep and recovery sleep after sleep deprivation were largely intact in older mice, suggesting that the well-described age-related changes in global sleep are unlikely to arise from a disruption of local network dynamics within the neocortex.

## Introduction

Aging is a strictly regulated biological process that is thought to provide selective advantages over attaining immortality and therefore increases the evolutionary success of a species. Aging refers to a variety of modifications that occur progressively as a function of preceding time spent alive, and can be observed at the molecular, cellular, and system levels, including in behavior and cognitive function (Burke and Barnes, 2006; Bishop et al., 2010; Kirkwood, 2010; Zoncu et al., 2010; Kourtis and Tavernarakis, 2011; Morrison and Baxter, 2012; Yeoman et al., 2012). Some of these processes are entirely physiological and reflect programmed aging, whereas others may reflect unwanted, normally inevitable but possibly preventable, consequences of various stressors encountered throughout life (Meyer et al., 1999; Enzinger et al., 2005).

Despite notable species differences, numerous studies in both humans and laboratory animals suggest that waking and sleep show systematic changes with aging; however, the biological significance of these changes is not well understood (Shiromani et al., 2000; Ohayon et al., 2004; Klerman and Dijk, 2008; Altena et al., 2010; Bano et al., 2011; Hasan et al., 2012; Klerman et al., 2013; Banks et al., 2015; Gu et al., 2015; Panagiotou et al., 2017). One possibility is that age-dependent changes in sleep are a reflection of anatomical or physiological changes, such as a loss of synaptic connectivity (Morrison and Baxter, 2012), altered Ca^2+^ homeostasis (Toescu and Vreugdenhil, 2010), a decline in the function of specific brain circuits (Porkka-Heiskanen et al., 2004; Wigren et al., 2009; Altena et al., 2010; Wang et al., 2011), increased susceptibility to cellular stress (Naidoo et al., 2008; Naidoo, 2009; Altena et al., 2010; Kourtis and Tavernarakis, 2011), or a progressive loss of circadian rhythmicity, such as a decline in the rhythmic output of the central circadian clock, the suprachiasmatic nucleus (Satinoff et al., 1993; Watanabe et al., 1995; Aujard et al., 2001; Biello, 2009; Colwell, 2011). It is also possible that the sleep changes that occur with aging may represent compensatory responses, in which sleep plays an active and increasingly important role in maintaining cellular homeostasis and optimal waking functions as the organism gets older. Consistent with this, sleep is known to play a crucial role in various restorative functions, including molecule biosynthesis, membrane repair, synaptic remodeling, and other cellular maintenance processes (Mackiewicz et al., 2008; Vyazovskiy and Harris, 2013; Xie et al., 2013; Tononi and Cirelli, 2014; Krueger et al., 2016).

While numerous studies have provided important insights into the global age-dependent alterations in sleep–wake architecture and electroencephalogram (EEG) across 24 h in mice (Vyazovskiy et al., 2006b; Hasan et al., 2012; Banks et al., 2015; Panagiotou et al., 2017), surprisingly little is known about the effects of aging on cortical neural activity. This is an important omission, as numerous studies suggest that the events occurring at a single neuron and local neuronal population level in the neocortex have important contributions to global sleep regulation (Vyazovskiy et al., 2009b, 2011; Grosmark et al., 2012; Fisher et al., 2016; Krueger et al., 2016; Rodriguez et al., 2016; Watson et al., 2016; Siclari and Tononi, 2017). It is well established that sleep–wake history is reflected in the level of EEG slow-wave activity (SWA, 0.5–4 Hz), which increases as a function of preceding wake duration and decreases during subsequent sleep, and is therefore used as a measure of sleep homeostasis (Borbély, 1982; Tobler and Borbély, 1986; Franken et al., 2001). Slow waves recorded with cortical EEG or local field potential (LFP) electrodes reflect the synchronous occurrence of population neuronal silence, corresponding to neuronal hyperpolarization or down-states within thalamocortical networks (Destexhe et al., 1999; Timofeev, 2013; Vyazovskiy and Harris, 2013; Crunelli et al., 2015; Neske, 2016). These so-called OFF periods become increasingly more frequent the longer the duration of wakefulness, and are most prominent during the initial deep non-rapid eye movement (NREM) sleep occurring immediately after periods of wakefulness. Despite being a defining feature of NREM sleep, locally occurring OFF periods may also be detected during wakefulness, especially after sleep deprivation (SD) (Vyazovskiy et al., 2009b, 2011), supporting the notion that sleep may be initiated at the level of local cortical networks (Krueger et al., 2016). While the local dynamics of slow waves and cortical neural activity during spontaneous sleep and after SD have been thoroughly characterized in young and adult animals, this has not previously been investigated in the context of aging.

Existing evidence suggests that aging may lead to specific changes in cortical activity during sleep. For example, a loss of gray and white matter with aging has been noted in humans (Coffey et al., 1992; Ge et al., 2002; Marner et al., 2003; Enzinger et al., 2005; Ziegler et al., 2012), and a consistent loss of hippocampal synaptic connections has been identified in rodents (Burke and Barnes, 2006, 2010; Morrison and Baxter, 2012). Both animal and human studies have shown aging to be associated with alterations in synaptic transmission and structural synaptic changes (Peters et al., 2008; Dumitriu et al., 2010; Morrison and Baxter, 2012; Petralia et al., 2014). Importantly, older animals have been shown to have larger synaptic field potentials in the hippocampus, suggesting that the reduction in synaptic contacts was partially compensated for by an increase in the electrical responsiveness of the remaining neurons (Barnes and McNaughton, 1980). In addition to these structural changes, previous studies have shown that aging leads to dramatic changes in global sleep characteristics, with sleep becoming more superficial or fragmented (Vyazovskiy et al., 2006b; Hasan et al., 2012; Wimmer et al., 2013; Banks et al., 2015). Although there is abundant evidence showing both structural and functional neuronal modifications with aging, it remains unknown whether the changes at the level of local cortical circuits are causally related to age-dependent changes in global sleep architecture.

This omission is particularly relevant, as it remains to be established whether aging is associated with a reduced homeostatic sleep need or instead diminishes the capacity to generate and sustain deep NREM sleep and the associated network oscillations (Klerman and Dijk, 2008; Cirelli, 2012; Mander et al., 2017). Because both sleep need and sleep depth are mechanistically and functionally related to the expression of SWA within local cortical networks (Massimini et al., 2004; Nir et al., 2011; Vyazovskiy and Harris, 2013; Neske, 2016), we set out to address the above question by chronically recording neural activity and LFPs in mice ∼5, 12, and 24 months of age. We hypothesized that, if age-related changes in sleep arise, at least in part, from a disruption of network activity within the neocortex, it should manifest in specific changes within local cortical circuits, such as reduced spiking activity, local slow waves and the underlying OFF periods, or altered neural dynamics at state transitions. Furthermore, we posited that, if aging primarily targets the capacity to produce an adequate homeostatic response to sleep loss at the level of local neuronal populations, this should reduce the occurrence of local cortical slow waves and OFF periods after SD. Surprisingly, we observed that aging had little effect on neural activity within local cortical networks, and that the local homeostatic response to SD, as measured by the occurrence of slow waves and OFF periods, was robust in older animals. Our findings suggest that the local cortical network mechanisms underlying sleep oscillations and sleep need are intact in older mice, and that the mechanisms underlying the profound global changes in sleep observed with aging are distinct from those responsible for local sleep regulation.

## Materials and Methods

### 

#### 

##### Experimental animals.

Recordings were performed in male C57BL/6J mice subdivided into three age groups: early adulthood (EA, 4.6 ± 0.3 months, *n* = 10), late adulthood (LA, 12.1 ± 0.2 months, *n* = 11), and older age (OA, 24.6 ± 0.4 months, *n* = 10). Although the exact correspondence between age in mice and humans remains a topic of debate, we estimate that the age of the older group in our study corresponded to ∼70 years in humans (Dutta and Sengupta, 2016). The number of animals used in this study was based on previous studies that investigated the effects of aging and sleep–wake history on sleep characteristics, including its amount, fragmentation, and EEG dynamics (Welsh et al., 1986; Colas et al., 2005; Hasan et al., 2012; Wimmer et al., 2013; Panagiotou et al., 2017). Mice were group housed until they underwent surgery. The implantation of microwire arrays can cause an immune response, which can deteriorate the electrophysiological signal and also destabilize the implant; we therefore opted not to perform longitudinal recordings. For this reason, mice were implanted shortly before they were required for surgery. On average, there were 29.4 ± 5.6, 22.8 ± 1.2, and 31.5 ± 3.7 d between the surgery date and baseline recording date, for EA, LA, and OA mice, respectively (Welch *F* test: *F*_(2,14.2)_ = 3.75, *p* = 0.049). At ∼2 weeks after surgery, mice were transferred to custom-made clear Plexiglas cages (20.3 × 32 × 35 cm), where they were individually housed with free access to a running wheel (RW; Campden Instruments, wheel diameter 14 cm, bars spaced 1.11 cm apart inclusive of bars) throughout the experiment and food available *ad libitum*. Cages were housed in ventilated, sound-attenuated Faraday chambers (Campden Instruments, two cages per chamber) under a standard 12:12 h light-dark cycle (lights on 0900, ZT0, light levels 120–180 lux). Room temperature and relative humidity were maintained at 22 ± 1°C and 50 ± 20%, respectively. All procedures conformed to the Animal (Scientific Procedures) Act 1986 and were performed under a UK Home Office Project License in accordance with institutional guidelines.

##### Surgical procedures and electrode configuration.

Surgical procedures were performed as previously described (Cui et al., 2014; Fisher et al., 2016), a summary of which is provided below. Surgical procedures were performed under aseptic conditions using isoflurane anesthesia (3%–5% induction, 1%–2% maintenance). One day before surgery, animals received dexamethasone (0.2 mg/kg, p.o.). Metacam (1–2 mg/kg, s.c.), dexamethasone (0.2 mg/kg, s.c.), and vetergesic (0.08 mg/kg, s.c.) were administered preoperatively. Before implantation, EEG screw electrodes were soldered to custom-made head-mount connectors (Pinnacle Technology) and unilaterally implanted into frontal (motor area: anteroposterior 2 mm, mediolateral 2 mm) and occipital (visual area, V1: anteroposterior 3.5–4 mm, mediolateral 2.5 mm) cortical areas. Reference and ground screw electrodes were implanted above the cerebellum and contralaterally to the occipital screw, respectively. Two single-stranded, stainless-steel wires were inserted on either side of the nuchal muscle to record electromyography. All screws and wires were secured to the skull using dental acrylic. Mice were also implanted with a polymide-insulated tungsten microwire array (Tucker-Davis Technologies) implanted into deep layers of the primary motor cortex. Because this is the first study that aimed to characterize the effects of aging on the cortical neural activity and local sleep homeostasis in mice, it was important to target an area that has been well studied in younger animals; therefore, we opted to perform LFP and multiunit activity (MUA) recordings from the frontal cortex (Vyazovskiy and Tobler, 2005; Vyazovskiy et al., 2011; Hajnik et al., 2013; Hayashi et al., 2015; Fisher et al., 2016). Furthermore, the frontal cortex was an obvious choice for investigating the effects of aging on the local slow wave homeostasis, as sleep EEG SWA has been shown to have frontal predominance in both rats and mice (Schwierin et al., 1999; Huber et al., 2000; Vyazovskiy et al., 2006a). The microwire array consisted of 16 channels (2 rows each of 8 wires), with a wire diameter of 33 μm, electrode spacing 250 μm, row separation L-R 375 μm, and tip angle of 45 degrees. Because of the curvature of the neocortex in the area of interest, the arrays were customized so that one row of electrodes was 250 μm longer than the other (Fisher et al., 2016). A 1 × 2 mm craniotomy was made using a high-speed drill with midpoint coordinates relative to bregma as follows: anteroposterior 1.5–2 mm, mediolateral 2 mm. Dorsal ventral coordinates were taken when the longer row of electrodes was touching the surface of the brain and the arrays were lowered below the pial surface into deep layers of the motor cortex. A two-component silicon gel (KwikSil; World Precision Instruments) was used to seal the craniotomy and protect the surface of the brain from the dental acrylic used to fix the array to the skull.

Animals were all monitored closely after surgery and scored daily for measures, such as grimace, appearance, natural behavior, and provoked behavior, until they were deemed to have returned to baseline for a minimum of 2 d. Mice were provided with appropriate analgesia as necessary (dexamethasone 0.2 mg/kg for 2 d and metacam 1–2 mg/kg for a minimum of 3 d). The animals were closely monitored after surgery for, on average, 6.1 ± 0.4, 6.7 ± 0.6, and 9.7 ± 0.9 d, for EA, LA, and OA mice, respectively. Therefore, OA animals took ∼3 d longer to recover from surgery compared with both EA and LA mice (Kruskal–Wallis with Mann–Whitney *post hoc*: χ_(2)_^2^ = 13.684, *P* < 0.0001; EA vs OA *p* < 0.0001, LA vs OA *p* = 0.004).

##### Signal processing, vigilance state scoring, and analysis.

A Tucker-Davis Technologies Multichannel Neurophysiology Recording System was used for data acquisition. Cortical EEG was recorded from frontal and occipital derivations. EEG, EMG, and LFP data were filtered between 0.1 and 100 Hz, amplified (PZ5 NeuroDigitizer preamplifier, Tucker-Davis Technologies), and stored on a local computer at a sampling rate of 256.9 Hz. Extracellular neuronal spike data were recorded from the microwire array at a sampling rate of 25 kHz (filtered between 300 Hz and 5 kHz). As an initial step, online spike sorting was performed using OpenEx software (Tucker-Davis Technologies) by manually applying an amplitude threshold for online spike detection to eliminate artifactual waveforms caused by electrical or mechanical noise. Spikes that exceeded this predefined threshold (>2× noise level, at least −25 μV) were stored as 46 samples (0.48 ms before, 1.36 ms after the threshold crossing) consisting of both voltage measures and time stamps. LFP, EEG, and EMG data were then resampled offline at a sampling rate of 256 Hz. Custom-written MATLAB scripts (The MathWorks) were used for signal conversion. Data were then transformed into European Data Format using open source Neurotraces software.

Recordings were subdivided into 4 s epochs and vigilance states scored offline by manual inspection of the signal (SleepSign, Kissei Comtec). Two EEG channels (frontal and occipital), EMG, two channels of MUA, and RW activity were simultaneously displayed to aid vigilance state scoring. Vigilance states were classified as waking (low-voltage, high-frequency EEG with a high level or phasic EMG activity), NREM sleep (presence of EEG slow waves, a signal of a high amplitude and low frequency), or REM sleep (low-voltage, high-frequency EEG with a low level of EMG activity). Vigilance state artifacts in at least one EEG or MUA recording channel, resulting from contamination by eating, drinking, or gross movements were also scored as artifacts so that they may be removed from appropriate analyses (percentage of total recording time: EA, 8.7 ± 3.1; LA, 8.8 ± 2.3; OA, 7.9 ± 3.5). After the data were scored, EEG and LFP power spectra were computed by an FFT routine for 4 s epochs (Hanning window), with a 0.25 Hz resolution (SleepSign, Kissei Comtec).

##### Experimental design and statistical analysis.

Because our primary aim was to investigate the effects of physiological healthy aging on spontaneous waking and sleep, rather than the effects of specific manipulations beyond the conventional SD, all experiments were performed under standard laboratory conditions, where mice were kept under a 12 h light:12 h dark cycle in their home-cage environment. Mice were transferred to the recording chambers and habituated to both the cage and recording cables for a minimum of 3 d before recording, until patterns of activity showed normal entrainment, as previously described (Cui et al., 2014; Fisher et al., 2016). On the day following the baseline recording, a 6 h SD was performed to investigate the effect of prolonged wakefulness on cortical activity. This was performed based on previous evidence that older mice may have a reduced capacity to respond to an increased sleep pressure (Hasan et al., 2012). SD was performed for 6 h starting at light onset using the well-established gentle handling technique (Vyazovskiy et al., 2002). Mouse behavior and polysomnographic recordings were constantly monitored; and when mice showed signs of sleepiness, the experimenter provided the animal with novel objects. This method is thought to mimic naturalistic waking conditions in an ethologically relevant manner, which is less stressful for the animals compared with other SD methods. Novel objects included cardboard, colorful plastic, and tissue paper. During SD, the animals repeatedly attempted to initiate sleep, and EA, LA, and OA mice slept, on average, 5.9 ± 0.9, 7.4 ± 2.7, and 12.3 ± 2.1 min, respectively, during the 6 h period.

For specific analyses, some mice were excluded for technical reasons, as stated in the figure legends. Data were analyzed using MATLAB and SPSS (IBM, released 2016; SPSS Statistics for Windows, version 24.0, IBM). In most cases, ANOVAs were used to identify differences between the three age groups. In cases where data failed homogeneity testing, a Welch *F* test with Games–Howell *post hoc* test was used instead. Where data failed normality testing, nonparametric Kruskal–Wallis tests, with Mann–Whitney *post hoc* tests were used. Critical *p* values were adjusted for multiple testing (*p*/number of tests), and only those values that reached the more stringent criteria are reported. For time course data, repeated-measures ANOVAs were used to identify differences. In a few cases where the animals did not sleep during a specific interval, the data for corresponding time points were estimated using a multiple imputation technique within SPSS (5 imputations used). Details of the specific statistical tests used are provided in the appropriate figure legends. Effect sizes were estimated by calculating Cohen's *d* (Lakens, 2013) for main findings. All values reported are mean ± SEM, unless explicitly stated.

##### Analysis of extracellular neuronal activity.

To investigate putative single-unit activity, we performed offline spike sorting, as previously described (Fisher et al., 2016). For spike sorting, we concatenated either a baseline light and dark period or a baseline light period and SD light period. An artifact removal procedure was used using custom written MATLAB scripts, to eliminate remaining artifactual waveforms. A principal component analysis was then performed on a segment of the spike waveform between the fifth and 35th time stamps as this segment is more informative about the overall spike waveform. After performing principal component analysis, each spike between samples 5 and 35 was described by 31 variables, each being a linear combination of the original sampling values. Clustering was then performed based on a *k*-means algorithm (Jolliffe, 2002), a partitioning method that aims to divide *n* observations into *k* clusters in which each observation belongs to the cluster with the nearest mean, serving as a prototype of the cluster. We used the *k*-means function in MATLAB, which was implemented according to Lloyd's algorithm (Lloyd, 1982). This approach requires the user to a priori select the number of clusters. All selected clusters were manually classified into *k* = 1, 2, 3, 4, or 5 clusters, and the best representation for that spike was visually classified based on the average spike waveform with SD, interspike interval distribution, the time course of peak-to-peak amplitude across the recording period, the corresponding time course of average firing rates, and the autocorellogram of the spike train. This was only performed for up to 5 clusters as it was unlikely that more than five clusters would occur in the same MUA electrode. To ensure cluster quality and stability, clusters were then further classified based on their signal-to-noise ratio, waveshape of the action potential, stability of the amplitude across time and ISI distribution histogram, and spurious or unstable clusters were excluded from the analysis as previously (Fisher et al., 2016).

##### Association between LFP slow waves and neuronal OFF periods.

**Figure 1.**
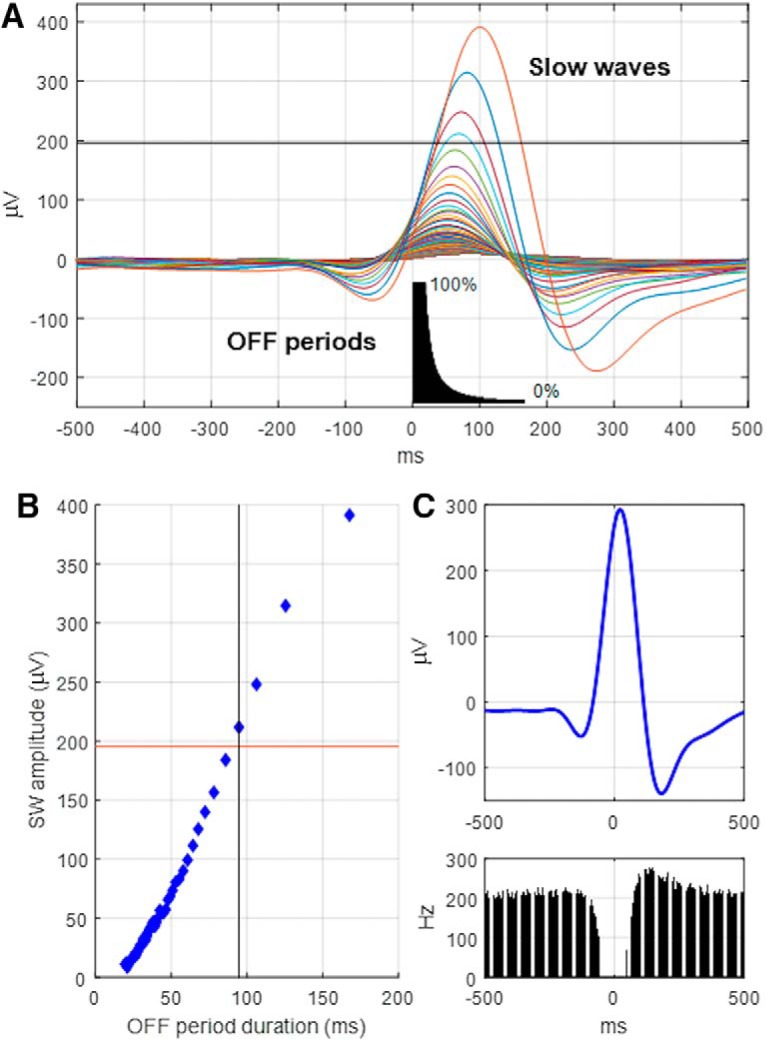
Algorithm for OFF period detection. ***A***, Average LFP slow waves calculated and plotted as a function of OFF period duration. All OFF periods were subdivided into 100 1% percentiles, and the corresponding average LFP signal aligned to the onset of an OFF period was calculated. Inset, The effect of lengthening the minimal duration of OFF periods on their average duration. Short OFF periods were not associated with noticeable changes in the LFP, whereas the highest-amplitude slow waves corresponded to the longest OFF periods. Horizontal line indicates the amplitude threshold used to define OFF periods in ***B***. ***B***, The relationship between the duration of OFF periods and the amplitude of corresponding average LFP slow waves in one individual animal. OFF periods were defined as periods of generalized network silence, which were associated with a slow wave at least 50% the amplitude of the largest slow waves (corresponding to the longest 1% of OFF periods). The corresponding thresholds are depicted as horizontal (slow wave amplitude) and vertical (minimal OFF period duration) lines. ***C***, The average LFP slow wave and corresponding profile of MUA centered on the midpoint of the OFF periods defined based on the above criteria.

Population OFF periods were defined as periods of total neuronal silence across all 16 electrodes, which were consistently associated with LFP slow waves. To detect OFF periods, we first concatenated all individual spikes and detected periods of silence lasting at least 20 ms and not exceeding 1000 ms. Next, we arranged all OFF periods into 100 1% percentiles and calculated the average LFP wave (mean between all recording channels) triggered to the onset of the corresponding OFF period (Fig. 1). This analysis revealed an exquisite sensitivity of the LFP to the occurrence of neuronal silence, and the size of the resulting LFP slow wave increased progressively as a function of longer OFF periods (Fig. 1). Subsequently, we defined OFF periods as periods of network silence, which were associated with a slow wave at least 50% the amplitude of the largest slow waves (100th percentile) and were therefore associated with the longest OFF periods in each animal (Fig. 1). To investigate the relationship between neuronal activity and LFP slow waves, two complementary approaches were used. First, the LFP signal was bandpass filtered between 0.5 and 4 Hz (stopband edge frequencies 0.3–8 Hz) with MATLAB filtfilt function exploiting a Chebyshev Type II filter design (Achermann and Borbély, 1997; Vyazovskiy et al., 2009b; Fisher et al., 2016), and waves were detected as positive deflections of the filtered LFP signal between two consecutive negative deflections below the zero-crossing. Only LFP waves with a peak amplitude larger than the median amplitude across all detected waves during baseline NREM sleep were included in subsequent analyses. Subsequently, all slow waves were aligned to their positive peak, and the corresponding profile of neuronal spiking was computed. Second, we detected OFF periods as described above and calculated the average LFP aligned to the onset of an OFF period. The incidence of OFF periods and slow waves as well as the duration of OFF periods were used in subsequent analyses to investigate age-dependent differences in neuronal network activity.

##### Neuronal “phenotyping” and vigilance-state dependency of cortical firing.

After spike sorting, we performed a characterization of the firing rates of the identified putative single units. The firing rates (i.e., the number of spikes per 1 s) of each neuron were determined for each 4 s epoch in artifact-free wake, NREM sleep, and REM sleep, and their distribution was plotted as a function of firing rate. This revealed a large variability in the vigilance-state specificity of neuronal firing across putative single units; with some neurons firing similarly across vigilance-states, whereas others fired at different frequencies according to vigilance-state. To visualize this variability across putative neurons, the predominant firing frequencies for each putative neuron was extracted from their respective histogram of the distribution of their firing rates. Neurons were then sorted according to their peak frequency and plotted in ascending order. The distribution width of the firing rates histogram was calculated separately for each state and age group as previously described (Fisher et al., 2016).

##### Histological verification of recording site.

At the end of the study, the electrode recording sites were confirmed using previously described histological methods (Fisher et al., 2016). Briefly, mice were transcardially perfused with 0.9% saline followed by 4% PFA solution. Brains were photographed to aid determination of electrode position, and then sectioned into 50 μm coronal slices using a freezing microtome. Sections were mounted onto slides and visualized using a fluorescence microscope. Before implantation, array electrodes were coated with a thin layer of DiI fluorescent dye (DiIC18(3), Invitrogen), to aid the identification of electrode tracts.

## Results

### The daily architecture of sleep is markedly altered in older mice

**Figure 2.**
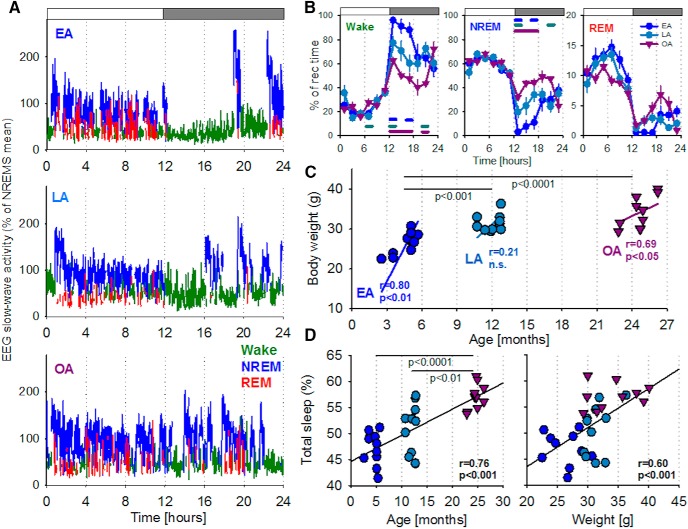
The global alterations of sleep with aging in mice. ***A***, Hypnograms of individual representative animals from each age group (EA, LA, OA). The 24 h profile of EEG SWA (EEG power between 0.5 and 4.0 Hz, represented as percentage of 24 h mean) recorded from the frontal cortex. Green represents Wake. Blue represents NREM sleep. Red represents REM sleep. Top, Bar represents the 12 h light and 12 h dark periods. ***B***, Time course of waking, NREM, and REM sleep during 24 h baseline day, shown in 2 h intervals. The amount of each vigilance state is represented as percentage of the total recording time. Data are mean ± SEM. EA, *n* = 10; LA, *n* = 11; OA, *n* = 10. Significant differences between ages are as follows: blue represents EA versus LA; cyan represents LA versus OA; purple represents EA versus OA. ***C***, The relationship between age and body weight across and within age groups. Filled symbols represent individual animals. Straight lines indicate linear regression lines separately for the three age groups. ***D***, The relationship of age (left) and body weight (right) with the amount of total sleep shown as percentage of recording time over 24 h. ***C***, ***D***, Data are mean ± SEM. EA, *n* = 10; LA, *n* = 11; OA, *n* = 10. *R* and *p* values correspond to Pearson's product moment correlation. Welch *F* test (Games–Howell *post hoc*) was used to compare age groups.

To investigate the age-dependent changes in cortical activity during waking and sleep, we performed continuous recordings of EEG, along with LFPs and extracellular neuronal activity (MUA) from deep cortical layers of the primary motor cortex (M1) of freely moving C57BL/6J mice. Consistent with previous reports (Welsh et al., 1986; Colas et al., 2005; Hasan et al., 2012; Wimmer et al., 2013; Panagiotou et al., 2017), we found that the older mice had a reduced amount of wakefulness (repeated-measures ANOVA: factor age: *F*_(2,28)_ = 20.6, *p* < 0.0001, age × time interval: *F*_(11,157)_ = 4.8, *p* < 0.0001), and an increased amount of NREM sleep (repeated-measures ANOVA: factor age: *F*_(2,28)_ = 21.0, *p* < 0.0001, age × time interval: *F*_(11,153)_ = 4.4, *p* < 0.0001), but not REM sleep (repeated-measures ANOVA: factor age: not significant, age × time interval: *F*_(12,169)_ = 3.8, *p* < 0.0001), during baseline recording days, particularly during the dark period (Fig. 2*A*,*B*). This resulted in a substantial increase in the total daily amount of sleep in older mice (percentage of recording time: EA, 47.0 ± 1.0%; LA, 50.6 ± 1.5%; OA, 57.1 ± 0.8%; Welch *F* test: *F*_(2,18)_ = 31.3, *p* < 0.0001; Fig. 2*D*). However, calculating the activity onset in each animal based on EEG/EMG defined wakefulness after lights off revealed that most animals were awake within minutes from dark onset, regardless of age (EA, 0.8 ± 0.3; OA, 1.1 ± 0.5; LA, 1.1 ± 0.3 min; not significant, Wilcoxon test).

Body weight also increased with age (EA, 26.4 ± 0.9; LA, 31.5 ± 0.6; OA, 34.1 ± 1.3; Welch *F* test: *F*_(2,17)_ = 15.7, *g* < 0.0001; Fig. 2*C*) and notably, both age and body weight showed a significant positive relationship with the total amount of sleep during 24 h (Fig. 2*D*). Because waking activities have an influence on the amount and distribution of sleep across 24 h (Vyazovskiy et al., 2006a; Vyazovskiy and Tobler, 2012; Fisher et al., 2016), we calculated the amount of RW activity in the three age groups. As expected, although all animals had free access to a RW throughout the experiment, younger mice used it more extensively than older mice (RW revolutions per hour of waking: EA, 485.8 ± 87.0; LA, 280.8 ± 84.7; OA, 38.1 ± 20.6; Welch *F* test: *F*_(2,15)_ = 15.0, *p* < 0.0001). Therefore, the pronounced age-dependent reduction in the amount of waking during the dark period (EA, 9.1 ± 0.3 h; LA, 8.0 ± 0.5 h; OA, 6.3 ± 0.2 h; Welch *F* test: *F*_(2,17)_ = 29.9, *p* < 0.0001) could merely reflect the reduced tendency for older animals to engage in continuous wheel running (Vyazovskiy et al., 2006a; Fisher et al., 2016). Consistently, the amount of waking was positively associated with RW activity across all ages (*r* = 0.84, *p* < 0.001). It should be noted, however, that causality and the directionality of the relationship between RW-activity and the capacity to sustain consolidated waking are difficult to determine using correlation analyses. Furthermore, because spontaneous wheel running is associated with substantial changes in cortical neuronal firing (Fisher et al., 2016) and may reflect a shift from preferentially goal-directed to automatic, habit-like behaviors (Vyazovskiy et al., 2017), the possibility remains that the global change in sleep–wake architecture with aging is mechanistically associated with specific changes in cortical circuit activity. If this were the case, it may be expected that such changes would be most readily detected in spontaneous patterns of network oscillations during sleep.

### The number of LFP slow waves and population OFF periods is increased with aging

**Figure 3.**
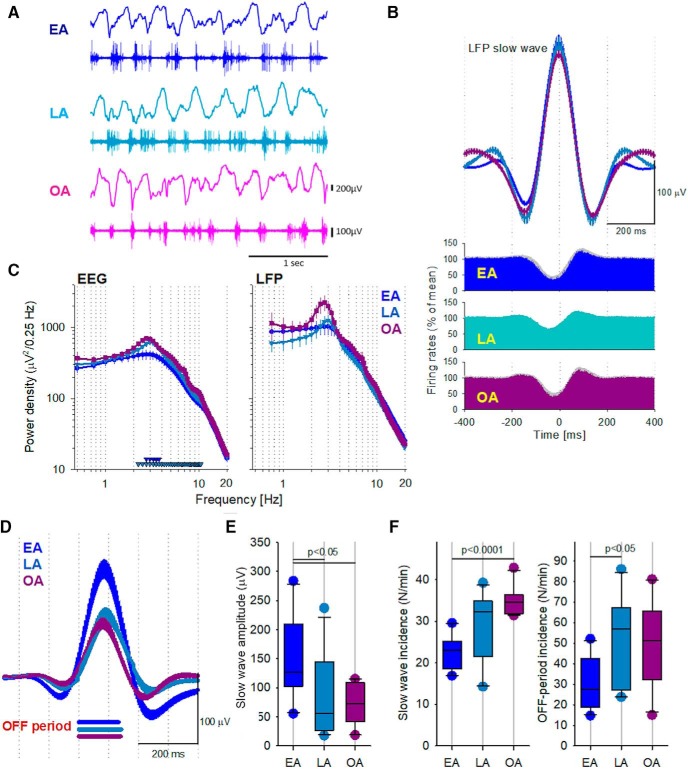
The relationship between LFP slow waves and cortical MUA in younger and older mice. ***A***, LFP (top) and MUA (bottom) traces from a representative LFP channel in representative animals from each age group. ***B***, Average LFP slow wave (top) and corresponding average MUA triggered by slow waves (plots below) in the three age groups. In all three ages, the positive LFP wave is associated with a clear-cut suppression of neuronal spiking. ***C***, Frontal EEG (left) and LFP (right) spectral power density during NREM sleep. Data are mean ± SEM. Bottom, Triangles represent frequency bins where EEG spectra differed significantly between the age groups (*p* < 0.05, unpaired *t* test on log-transformed values; top: EA vs LA; bottom: EA vs OA). ***D***, Average LFP slow wave triggered by the onset of generalized neuronal silence (an OFF period) across all recorded neurons. Despite the average duration of OFF periods being similar between ages, the amplitude of the resulting slow wave was higher in EA animals, compared with LA and OA mice. ***E***, The effect of aging on the amplitude of the average LFP slow wave triggered by population OFF periods (as shown in ***D***). EA, *n* = 10; LA, *n* = 11; OA, *n* = 10. A one-way ANOVA (Tukey *post hoc* test) was used to compare age groups. ***F***, The effect of aging on the incidence of slow waves and OFF periods during baseline NREM sleep. EA, *n* = 10; LA, *n* = 11; OA, *n* = 10. For slow wave incidence, a Welch *F* test (Games–Howell *post hoc*) was used to compare age groups. For OFF period incidence, a one-way ANOVA (Tukey *post hoc*) was used to compare age groups.

As is well established, EEG and LFP slow waves during NREM sleep are associated with characteristic changes in the membrane potential of cortical neurons, which give rise to synchronous transitions across cortical neuronal populations between periods of activity (ON periods) and silence (OFF periods) (Destexhe et al., 2007; Nir et al., 2011; Vyazovskiy and Harris, 2013; Lemieux et al., 2015; Rodriguez et al., 2016). Based on this, our first question was to determine whether these neuronal correlates of LFP slow waves are altered in older animals. In contrast to previous human studies showing that EEG slow waves are markedly reduced with aging, we observed that the LFP and MUA signals were similar across age groups, and visual inspection of the signals alone was not sufficient to differentiate between the age groups (Fig. 3*A*; representative recordings of LFP and MUA signals from individual mice from EA and OA age groups are shown in Movies 1, 2). In all three ages, LFP traces during NREM sleep were characterized by pronounced positive slow waves associated with an unequivocal suppression of MUA in the corresponding channels, typically encompassing most, if not all, recording channels. Calculation of the average MUA triggered by individual slow waves confirmed this and revealed a suppression of neural spiking in association with slow waves in all age groups (Fig. 3*B*). Consistent with a recent report (Panagiotou et al., 2017), LA and OA mice were found to have significantly higher EEG spectral power density in slow frequencies (<10 Hz) compared with young controls (Fig. 3*C*), yet the difference did not reach significance when we compared spectral power of the LFP (Fig. 3*C*). This contradicts human studies (Dijk et al., 1989; Landolt and Borbély, 2001; Mander et al., 2013), which instead report a decrease in EEG spectral power in the SWA frequency range with age.

Movie 1. Example raw electrophysiological signals recorded from a representative EA mouse during NREM sleep. Four channels of LFPs and corresponding MUA (pNeu) are shown; 5 s of recording is shown at any given time, at a playback speed 2× normal. Note the occurrence of synchronous silent (OFF) periods in the MUA, corresponding to slow waves in the LFP. 10.1523/JNEUROSCI.2513-17.2018.video.1

Movie 2. Example raw electrophysiological signals recorded from a representative OA mouse during NREM sleep. Four channels of LFPs and corresponding MUA (pNeu) are shown; 5 s of recording is shown at any given time, at a playback speed 2× normal. Note the occurrence of synchronous silent (OFF) periods in the MUA, corresponding to slow waves in the LFP, similar to those seen in the younger age groups. 10.1523/JNEUROSCI.2513-17.2018.video.2

Because EEG and LFP are influenced by both distant and local sources, reflect volume conduction, and may also be entrained by oscillations occurring elsewhere in the cortex (Sirota et al., 2008), we next focused on investigating the activity of cortical neurons. First, to assess the relationship between LFP slow waves and the underlying local neural activity, all detected OFF periods were aligned to their onset and the corresponding LFP signals were averaged. LFP slow waves were exquisitely sensitive to the duration of corresponding OFF periods in all ages, with the amplitude of slow waves progressively increasing with a lengthening of OFF period duration (Fig. 1). Notably, the average amplitude of the slow wave triggered by OFF periods was reduced in both LA and OA mice compared with EA mice (ANOVA: *F*_(2,28)_ = 4.9, *p* = 0.015; Fig. 3*D*,*E*), despite similar average OFF period durations (EA, 133.9 ms; LA, 141.9 ms; OA, 140.0 ms; not significant). This suggests that the spiking activity and silence of individual cortical neurons may, to some extent, be uncoupled from the slow network LFP oscillation in older animals. To further address this, we calculated the average incidence of LFP slow waves and population OFF periods during an undisturbed baseline 12 h light period. Interestingly, the incidence of both increased significantly with age (slow wave incidence: Welch *F* test: *F*_(2,18)_ = 24.9, *p* < 0.0001, OFF period incidence: one-way ANOVA: *F*_(2,28)_ = 3.9, *p* = 0.031; Fig. 3*F*), with the majority of effect sizes corresponding to large effects as per Cohen's convention (Lakens, 2013) (slow wave incidence: *d* = −3.20, *d* = −0.84, *d* = −0.96; OFF period incidence: *d* = −1.15, *d* = −1.17, *d* = 0.07; for EA vs OA, EA vs LA, and LA vs OA, respectively).

### The response of local cortical network activity to SD is intact in older animals

**Figure 4.**
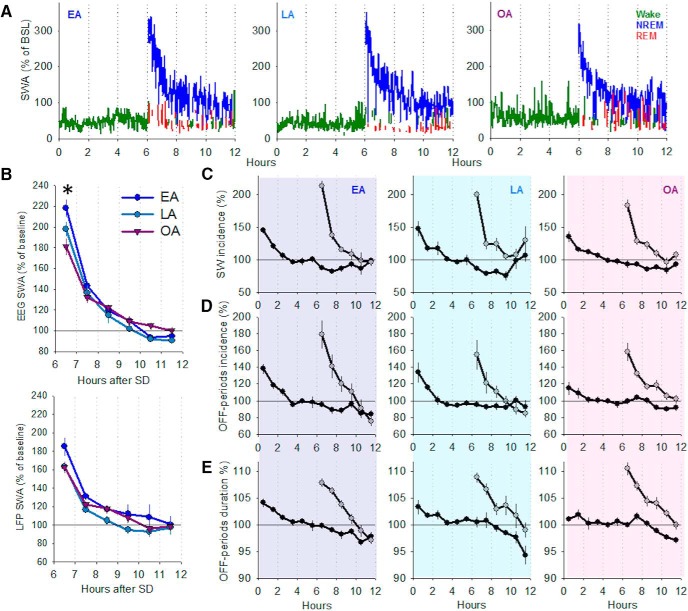
Effects of SD on cortical slow waves and OFF periods in older mice. ***A***, Representative hypnograms of individual animals from each age group (EA, LA, OA). The 12 h profile of EEG SWA (EEG power between 0.5 and 4.0 Hz, represented as percentage of baseline 24 h mean), recorded in the frontal cortex, is color-coded according to the vigilance state: green represents Wake; blue represents NREM sleep; red represents REM sleep. SD was performed for 6 h from light onset. After SD, a robust increase in EEG SWA is evident in all 3 animals, which is followed by a progressive decline during the subsequent recovery period. ***B***, Time course of EEG (top) and LFP (bottom) SWA during 6 h period after 6 h SD. Data are mean ± SEM. EA, *n* = 7; LA, *n* = 5 or 6; OA, *n* = 9. The time course of the decline in SWA was not significantly different between age groups for either the frontal EEG of LFP (repeated-measures ANOVA). *Significant difference between EA and OA mice in the first hour after SD (*p* = 0.01, one-way ANOVA with Tukey *post hoc* test). ***C***, The effect of SD on the incidence of LFP slow waves for a 12 h baseline period and the 6 h period following SD. Values are shown in 1 h intervals. Data are mean ± SEM. EA *n* = 7; LA *n* = 7; OA *n* = 10. Repeated-measures ANOVAs used to identify age differences during baseline and recovery after SD. One-way ANOVAs used to identify age differences in the initial rebound after SD. ***D***, ***E***, The same analyses were performed as in ***C***, but for OFF period incidence and duration.

It has been well described that, in laboratory rodents, EEG SWA during NREM sleep decreases progressively during the light period and is higher after prolonged spontaneous wakefulness or SD (Tobler and Borbély, 1986; Vyazovskiy et al., 2009b, 2011). These dynamics are associated with a higher incidence of slow waves and neuronal OFF periods, which are considered markers of increased physiological sleep pressure at the network level (Vyazovskiy et al., 2007, 2009b; Rodriguez et al., 2016). Therefore, we next investigated the dynamics of both measures of network activity across the baseline 12 h light period, when mice are predominantly asleep, and then performed SD the following day to determine the effect of prolonged wakefulness on these measures. We found that the incidence of both slow waves and OFF periods as well as the duration of OFF periods decreased significantly across the baseline light period in all three age groups (repeated-measures ANOVAs: slow wave incidence factor time interval, *F*_(5,96)_ = 19.6, *p* < 0.0001, Fig. 4*C*; OFF period incidence factor time interval, *F*_(4,74)_ = 12, *p* < 0.0001, Fig. 4*D*; OFF period duration factor time interval, *F*_(6,116)_ = 13.6, *p* < 0.0001, Fig. 4*E*); however, only the time course of OFF period incidence was significantly different between age groups (repeated-measures ANOVA: factor age *F*_(2,21)_ = 7, *p* = 0.005; EA vs LA, *p* = 0.009; LA vs OA, *p* = 0.01).

Previous studies have suggested that older humans and rodents have a reduced capacity to generate a rebound in SWA in response to SD, with enhanced age-dependent differences found to occur after SD (Münch et al., 2004; Lafortune et al., 2012; Wimmer et al., 2013). Interestingly, in our study, we observed a robust increase in SWA after SD in all three ages (individual plots, Fig. 4*A*), with no age differences identified for the time course of SWA in the 6 h of recovery sleep after SD, for either the frontal EEG or LFP (Fig. 4*B*). The initial levels of SWA in the frontal EEG derivation were attenuated in OA mice (EA, 218.1 ± 8.5; LA, 197.8 ± 7.3; OA, 181.1 ± 8.3; ANOVA factor age, *F*_(2,18)_ = 5.4, *p* = 0.015; Fig. 4*B*), whereas no significant age differences were identified for the LFP SWA. Effect sizes were calculated for the first hour after SD, a time point at which the main compensatory effect of SD is observed. Large effect sizes were observed for comparisons of EA versus OA (EEG, *d* = 1.55; LFP, *d* = 1.01) and EA versus LA (EEG, *d* = 1.00; LFP, *d* = 1.00), whereas small or medium effect sizes were observed for LA versus OA comparisons (EEG, *d* = 0.74; LFP, *d* = 0.04). The incidence of slow waves and OFF periods as well as the duration of OFF periods were significantly higher after SD compared with the same 6 h of the baseline recording day in all three ages (repeated-measures ANOVA factor day: slow wave incidence, *F*_(1,42)_ = 108.6, *p* < 0.0001, Fig. 4*C*; OFF period incidence, *F*_(1,42)_ = 29.9, *p* < 0.0001, Fig. 4*D*; OFF period duration, *F*_(1,42)_ = 84.5, *p* < 0.0001, Fig. 4*E*). This increase after SD was not significantly different between age groups (ANOVA, factor age; slow wave incidence, *F*_(2,42)_ = 0.185, *p* = 0.832, Fig. 4*C*; OFF period incidence, *F*_(2,42)_ = 0.99, *p* = 0.38, Fig. 4*D*; OFF period duration, *F*_(2,42)_ = 2.776, *p* = 0.074, Fig. 4*E*). Effect sizes were then calculated for the first hour after SD. For slow wave incidence, a large effect size was detected for EA versus OA comparisons (*d* = 1.02), whereas medium effect sizes were detected for EA versus LA (*d* = 0.62) and LA versus OA (*d* = 0.62) comparisons. For OFF period incidence, medium effect sizes were detected for EA versus OA (*d* = 0.51) and EA versus LA (*d* = 0.52) comparisons, whereas LA versus OA comparisons only revealed a small effect size (*d* = −0.09). In contrast, OFF period duration only had small effect sizes for EA versus LA (*d* = 0.39) and LA versus OA (*d* = −0.46) comparisons, whereas the EA versus OA comparison had a medium to large effect size (*d* = −0.75). All three age groups showed a comparable gradual decrease in slow wave incidence, OFF period incidence, and OFF period duration over the 6 h recovery after SD (repeated-measures ANOVA factor time interval: slow wave incidence, *F*_(3,53)_ = 61.3, *p* < 0.0001, Fig. 4*C*; OFF period incidence, *F*_(2,35)_ = 50.0, *p* < 0.0001, Fig. 4*D*; OFF period duration, *F*_(3,56)_ = 54.1, *p* < 0.0001, Fig. 4*E*; factor time interval × age: slow wave incidence, *F*_(5,53)_ = 1.6, *p* = 0.179, Fig. 4*C*; OFF period incidence, *F*_(3,35)_ = 1.7, *p* = 0.142, Fig. 4*D*; OFF period duration, *F*_(5,56)_ = 0.654, *p* = 0.67, Fig. 4*E*). Therefore, in our study, the response to SD was not markedly different between age groups, apart from in the initial level of EEG SWA in the frontal derivation. The possibility remains that the pronounced baseline differences in the amount of sleep between ages could influence the homeostatic response after SD. Indeed, correlation analyses revealed a negative relationship between the amount of NREM sleep during the baseline dark period before SD and the magnitude of the increase in the incidence of slow waves and OFF periods (*p* < 0.05 for both).

It has previously been suggested that the higher absolute EEG SWA observed in older animals may reflect higher sleep pressure (Panagiotou et al., 2017). One of the established markers of higher sleep propensity is a faster buildup of SWA within NREM sleep episodes. To address whether this increase is present in older animals, we quantified the incidence of LFP slow waves and OFF periods in the first 2 min after the onset of an NREM sleep episode, during both baseline recordings and after SD. Interestingly, while EA mice showed a robust increase in both parameters after SD, this was largely attenuated or absent in LA and OA mice (percentage change in SW incidence relative to baseline: EA 20.3 ± 5.5; LA 2.0 ± 5.5; OA −3.9 ± 4.7, one-way ANOVA factor age *F*_(2,21)_ = 5.8, *p* = 0.01; OFF period incidence: EA 50.1 ± 14.1; LA −7.6 ± 7.6; OA −2.3 ± 18.3; Kruskal–Wallis test χ_(2)_^2^ = 8.214, *p* = 0.014, with a mean rank score of 18.86 for EA, 10.86 for LA, and 9.2 for OA mice). One possibility is that this may reflect a reduced capacity to engage in deeper NREM sleep with aging, perhaps due to increased neuronal activity or excitability during NREM sleep (Klerman and Dijk, 2008). On the other hand, as the older animals had a higher absolute number of slow waves and OFF periods (Fig. 3*F*), there may be a ceiling effect taking place, in which no further increase is possible.

### The dynamic repertoire of the activity of single neurons during wake and sleep is largely stable across the life span

**Figure 5.**
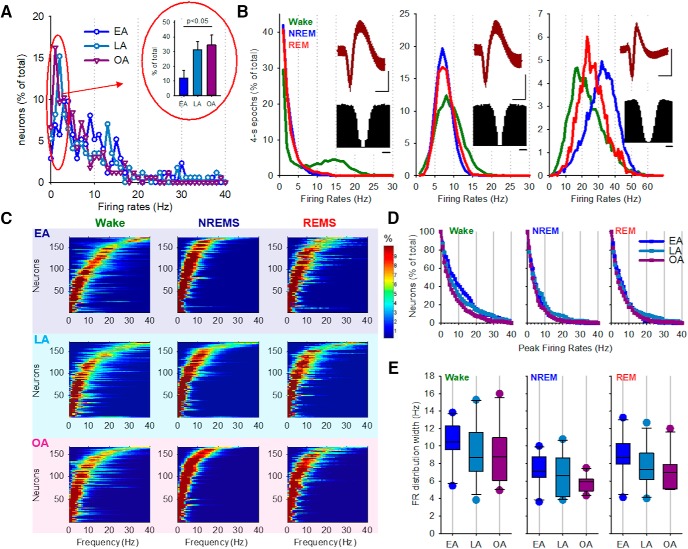
Aging and the vigilance state dependence of cortical neuronal firing. ***A***, The distribution of the firing rates of all putative individual neurons across all vigilance states, plotted as a proportion of the total number of neurons. On average, 17.4 ± 2.9, 15.6 ± 1.7, and 16.7 ± 2.5 putative single neurons per mouse contributed to the analysis for EA, LA, and OA mice, respectively. Inset, The proportion of neurons that fired at slow rates (0–3 Hz), which was significantly different between age groups (one-way ANOVA, Tukey *post hoc* test). ***B***, Distribution of firing rates across 4 s epochs expressed as a percentage of the total number of epochs, for three representative individual putative single units. Green represents Wake; blue represents NREM sleep; red represents REM sleep. Subplots represent the corresponding average spike waveform (± SD) and autocorrelogram. Individual neurons are highly variable with regards to their vigilance-state specific firing. ***C***, The predominant firing rates for all putative neurons were extracted from the distribution histograms (representative examples shown in ***B***), sorted by their peak firing rate, and plotted in ascending order for each vigilance state separately. Neurons did not fall into distinct categories based on their firing rates but rather formed a continuum in which all possible peak firing rates could be observed. ***D***, The proportion of neurons discharging at a specific frequency is shown separately for Wake, NREM sleep, and REM sleep. ***E***, Mean firing rate distribution widths for Wake, NREM sleep, and REM sleep are shown for the three age groups as follows: EA, *n* = 10; LA, *n* = 11; OA, *n* = 10. No statistical differences between age groups were identified (one-way ANOVA).

The age-dependent changes in neuronal population activity we observed may arise either at a single-cell level or reflect larger scale processes, such as global neuromodulation. Therefore, next we investigated the effect of aging on the vigilance state-specific discharge of individual cortical neurons. On average, of 16 microwire channels, 9.2 ± 1.0, 9.3 ± 1.0, and 9.2 ± 1.0 channels showed robust MUA in EA, LA, and OA animals, respectively, and subsequent spike sorting resulted in 17.4 ± 2.9, 15.6 ± 1.7, and 16.7 ± 2.5 putative single units detected per animal, which was not significantly different between ages. Previous studies suggest that the distribution of firing rates between individual neurons is best characterized by a log-normal distribution, which renders calculating the mean firing rate or averaging between individual neurons inappropriate (Watson et al., 2016). Therefore, we plotted the distribution of firing rates across neurons, which revealed that the older groups of mice had a somewhat higher proportion of slow spiking neurons (Fig. 5*A*). However, it is possible that this may be related to the higher amount and presumably intensity of waking in EA animals, compared with LA and OA mice.

It should be noted that the distribution of firing activity within individual neurons is also highly variable (Fisher et al., 2016) and often deviates from normality (Fig. 5*B*). This therefore also poses a problem for calculating average firing rates within individual neurons. Previous studies suggest that cortical neurons change their firing characteristics depending on the global behavioral state, although a great variability between individual neurons and cortical regions has been noted (Hobson and McCarley, 1971; Vyazovskiy et al., 2009b; Fisher et al., 2016; Niethard et al., 2016). For example, it is typically observed that neurons fire at a higher rate during waking and REM sleep compared with NREM sleep (Vyazovskiy et al., 2009b), although this is not always the case, and this may be influenced by the recording technique or by the cortical region being recorded (Hobson and McCarley, 1971; Niethard et al., 2016). The firing rates of cortical neurons are determined by their electrophysiological characteristics, connectivity pattern, ongoing behavior, or preceding sleep–wake history (O'Keefe and Dostrovsky, 1971; Ascoli et al., 2008; Poulet and Petersen, 2008; Kropff et al., 2015; McGinley et al., 2015; Fisher et al., 2016). We therefore performed neuronal “phenotyping” by calculating, for each putative single neuron, the distribution of their firing rates across all 4 s epochs of NREM sleep, REM sleep, and waking separately (Fig. 5*B*,*C*). Individual neurons were highly idiosyncratic with respect to their state-dependent firing (Fig. 5*B*), and so we hypothesized that neurons could be subdivided into distinct categories based on the distribution of their spiking activity during waking and sleep, as is traditionally done for subcortical areas where sleep- or wake-active neurons have been identified (Jones, 2005). To this end, we determined the peak frequency for each putative single unit based on a histogram of its firing rates (examples shown in Fig. 5*B*) and then plotted the histograms separately for each vigilance state (Fig. 5*C*). Interestingly, we observed that cortical neurons could not be subdivided into distinct categories based on their firing rates, but instead they formed a continuum, where virtually any firing phenotype could be observed within each vigilance state (Fig. 5*C*). Furthermore, the distribution of cortical neurons as a function of their firing rates was visually indistinguishable between ages (Fig. 5*C*). On average, 13.7 ± 3.3%, 15.9 ± 6.6%, and 14.5 ± 4.5% of all putative single units, in EA, LA, and OA mice, respectively, discharged at a higher average firing rate during sleep (including both NREM and REM sleep) compared with waking, and the proportion of such “sleep-active” cortical neurons was similar between ages. However, plotting the distribution of individual neurons as a function of their spiking activity revealed that during wake faster spiking neurons were more common in EA mice (Fig. 5*D*). This effect is likely accounted for by a higher amount of waking and increased levels of arousal in general in younger animals. It should be noted that, although during wheel running the older animals did not attain the same speed as the younger animals, we observed a negative relationship between the firing rates of individual putative neurons and running speed in all three age groups, consistent with our previous study performed in younger animals (Fisher et al., 2016). Furthermore, the distribution of neurons as a function of their spiking activity during spontaneous NREM and REM sleep also did not reveal any noticeable differences between ages (Fig. 5*D*). Finally, as previous studies have shown that the distribution width of the firing rates during waking is influenced by behavior (Fisher et al., 2016), we next addressed whether this is also different between ages. No statistical differences were observed in the firing rate distribution widths between age groups, for any vigilance state (Fig. 5*E*). Therefore, as mice get older, the overall composition of fast-spiking and slow-spiking, as well as “sleep-active” and “wake-active” cortical neurons appears stable, and neurons largely retain their vigilance state-dependent firing profile. This suggests that the dynamic repertoire of the activity of single neurons during both waking and sleep is stable across the lifespan. This observation is interesting as it suggests an intriguing possibility that the mechanisms underlying the gross alteration of the daily sleep–wake distribution are distinct from those implicated in the regulation of local cortical states.

### Persistently firing cortical “sleep-active” neurons are more abundant in older animals

**Figure 6.**
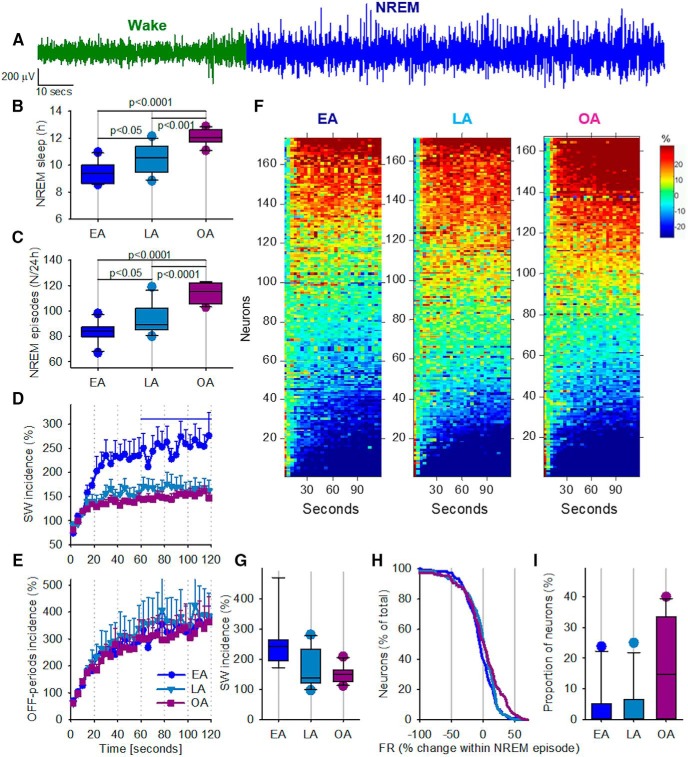
Intraepisodic dynamics of cortical firing at the transition to NREM sleep. ***A***, Representative example of a cortical LFP recording at the transition from waking to NREM sleep. ***B***, The effect of aging on the total amount of NREM sleep during 24 h. EA, *n* = 10; LA, *n* = 11; OA, *n* = 10. One-way ANOVA (Tukey *post hoc* test) was used to compare age groups. ***C***, The effect of aging on the total number of NREM sleep episodes during 24 h. EA, *n* = 10; LA, *n* = 11; OA, *n* = 10. One-way ANOVA (Tukey *post hoc* test) was used to compare age groups. ***D***, The time course of relative LFP slow wave incidence during the first 2 min after the onset of NREM sleep episodes. Values are percentage of the first 12 s. Data are mean ± SEM. Line indicates the second minute of NREM sleep, which undergoes further analysis in ***G***. ***E***, The same analysis as in ***D***, but for the incidence of OFF periods. ***F***, The dynamics of firing rates during the first 2 min after the onset of an NREM sleep episode are shown for all individual putative single units across all animals. The neurons are sorted as a function of the relative firing rates attained during the second minute after the episode onset. ***G***, Slow wave incidence during the second minute of NREM sleep episode shown as percentage of the corresponding value during the first 12 s after the onset of NREM sleep episode. EA, *n* = 9; LA, *n* = 11; OA, *n* = 10. A nonparametric Kruskal–Wallis test with Mann–Whitney *post hoc* test (exact, two-tailed) was used to test for significant differences between age groups. *Post hoc* tests for EA versus OA and LA versus OA gave *p* values of 0.033 and 0.037, respectively; this was not significant after correcting for multiple testing (critical value *p* = 0.0167). ***H***, Distribution of all putative neurons as a function of the change in their firing frequency within NREM sleep episodes. ***I***, The proportion of neurons, which show at least a 30% increase in their rate of discharge during the second minute after NREM sleep onset relative to the first 12 s after the initiation of corresponding NREM sleep episodes. A nonparametric Kruskal–Wallis test with Mann–Whitney *post hoc* test (exact, two-tailed) was used to test for significant differences between age groups. EA versus OA: *U* = 4, *z* = −3.348, *p* < 0.0001. *Post hoc* testing for EA versus LA gave a *p* value of 0.031; however, this was not significant after correcting for multiple testing (critical value *p* = 0.0167).

We next asked whether the age-dependent changes in sleep and wake could be reflected in the neuronal dynamics at vigilance state transitions, such as at the onset of individual NREM sleep episodes (Fig. 6*A*). We suggest that this might be the case for two reasons. First, the overall amount of NREM sleep was substantially higher in older animals (one-way ANOVA: *F*_(2,28)_ = 23.7, *p* < 0.0001; Fig. 6*B*), whereas the number of transitions into NREM sleep was also significantly higher in older animals (one-way ANOVA *F*_(2,28)_ = 26.9, *p* < 0.0001; Fig. 6*C*). Because it has been hypothesized that sleep is a cortical circuit phenomenon that may be initiated by local networks (Pigarev et al., 1997; Krueger et al., 2008; Hinard et al., 2012; Lemieux et al., 2014; Sanchez-Vives and Mattia, 2014), it is reasonable to assume that the neuronal dynamics involved in sleep initiation may be different depending on the level of local sleep propensity. Previous studies showed that, as an animal transitions into NREM sleep, overall firing rates become progressively slower, possibly due to the occurrence of neuronal silent periods (Vyazovskiy et al., 2009b). At the same time, it has previously been shown that SWA, as well as the amplitude of slow waves, increases progressively during the first 1–2 min after NREM sleep onset, reflecting a progressive increase in sleep depth (Vyazovskiy et al., 2009a; Cui et al., 2014). Consistently, we observed an increase in relative slow wave incidence across the first 2 min of an NREM sleep episode, which was larger and more rapid in EA mice compared with older animals (Fig. 6*D*). One possible explanation for older animals having an attenuated buildup of slow wave incidence is that the absolute incidence of both slow waves and OFF periods was higher in older animals throughout NREM sleep (Fig. 3*F*). However, curiously, the gradual increase in the number of OFF periods during the first 2 min of NREM sleep episodes did not differ noticeably between ages (Fig. 6*E*).

We next plotted the neuronal firing activity of all individual putative neurons across all animals during the first 2 min after the onset of an NREM sleep episode. Once again, neurons did not fall into distinct categories based on their dynamics in the first few minutes after NREM sleep onset. Instead, we observed that, even within a small cortical network, the recorded neurons presented an entire spectrum of possible changes: with some neurons showing a progressive increase in spiking in the initial minutes after sleep onset, whereas other neurons did not change their spiking activity or showed a pronounced decrease (Fig. 6*F*). It was apparent, however, that, in OA mice, a somewhat larger proportion of neurons increased their spiking activity in the initial few minutes after the onset of NREM sleep episodes (Fig. 6*F*), which is consistent with the observation that OA mice had a reduced relative increase in the incidence of slow waves as sleep progressed into the second minute (Kruskal–Wallis H test, χ_(2)_^2^ = 9.979, *p* = 0.007, with a mean rank age score of 23.22 for EA, 12.73 for LA, and 11.6 for OA mice; Fig. 6*G*). This may suggest that, even within individual NREM sleep episodes, the progression from a relatively more superficial sleep to a deeper sleep is attenuated in older animals. This was confirmed by the finding that older animals have more intense relative neuronal spiking in the second minute of NREM sleep episodes (mean rank age scores 12.9 for EA, 13.86 for LA, and 21.45 for OA mice, Kruskal–Wallis H test, χ_(2)_^2^ = 6.4, *p* = 0.04; Fig. 6*H*,*I*). Effect sizes calculated for the proportion of neurons that increased firing by at least 30% at the onset of NREM sleep (Fig. 6*I*) revealed large effect sizes for EA versus OA (*d* = −1.09) and LA versus OA (*d* = −1.06), with a small effect size for EA versus LA (*d* = −0.07) comparisons. We hypothesize that this could affect sleep intensity, making sleep relatively more superficial and easier to disrupt, or it could reflect local state instability (Doran et al., 2001; Parrino et al., 2012), which may interfere with the restorative functions of sleep. However, the high incidence of cortical neurons that increased their firing rates across NREM sleep episodes is somewhat surprising and appears to contradict the occurrence of OFF periods, which are associated with reduced neuronal spiking but were not altered or even enhanced, in absolute terms, in older animals.

### Aging does not affect the dynamics of cortical firing at NREM-REM sleep transitions

**Figure 7.**
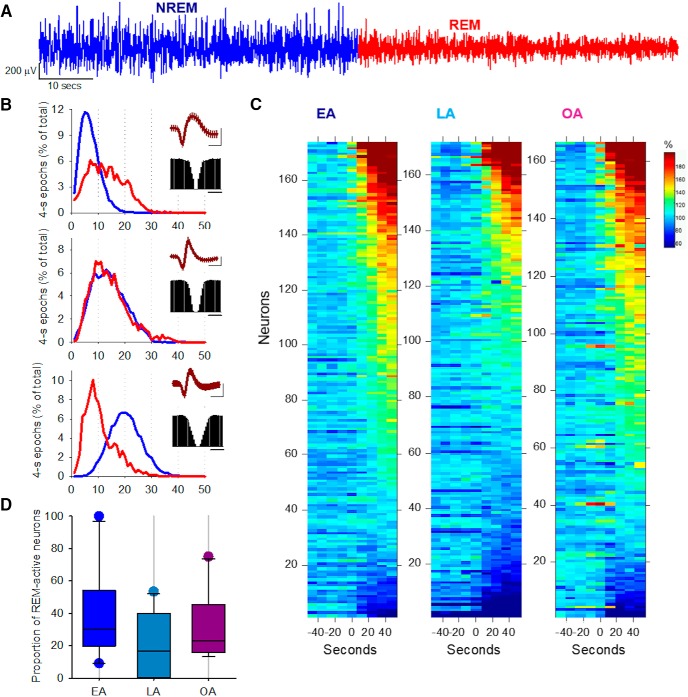
The effects of aging on the neuronal dynamics at the transition from NREM to REM sleep. ***A***, Representative example of cortical LFP at the transition from NREM sleep to REM sleep. On average, 51.9 ± 3.7, 45.8 ± 2.0, and 45.3 ± 2.5 N-R transitions contributed to the analysis below for EA, LA, and OA animals, respectively. ***B***, Distribution of firing rates across 4 s epochs in NREM sleep (blue) and REM sleep (red) shown for three representative individual putative single units. Each subplot represents the average spike waveform (± SD) and the autocorrelogram. Individual neurons show great diversity in their state dependent firing within sleep. ***C***, The dynamics in firing rates during the last minute of NREM sleep and the first minute of subsequent REM sleep are shown for all individual putative single units across all animals. The neurons are sorted as a function of their relative firing rates attained during REM sleep. ***D***, The proportion of putative single neurons, discharging, on average, at a higher rate during REM sleep compared with NREM sleep, expressed as a percentage of all neurons in the three age groups: EA, *n* = 10; LA, *n* = 11; OA, *n* = 10. One-way ANOVA did not identify any significant differences between age groups.

Transitions between NREM and REM sleep (Fig. 7*A*) are characterized by profound changes in cortical EEG, LFPs, and neuronal activity (Vyazovskiy et al., 2009b; Funk et al., 2016; Niethard et al., 2016), as well as marked changes across several subcortical neuromodulatory systems (Fort et al., 2009; Van Dort et al., 2015; Weber et al., 2015). Once again, we observed substantial variability in the response of individual neurons to transitions between NREM and REM sleep (Fig. 7*B*,*C*), whereas the overall number of “REM-sleep active” cortical neurons was not different between ages (Fig. 7*D*). Interestingly, a substantial proportion of neurons exhibited very similar patterns of activity between NREM and REM sleep. This supports the notion that both sleep states share important common characteristics (Funk et al., 2016), and further suggests that state-dependent changes in cortical activity during sleep are not affected markedly by the aging process, at least in the motor cortex of mice.

## Discussion

Intensive research over the past few decades revealed numerous effects of aging on sleep amount and architecture, as well as on specific sleep oscillations in both humans and animals (Dijk et al., 1989; Bliwise, 1993; Landolt and Borbély, 2001; Carrier et al., 2011; Cirelli, 2012; Hasan et al., 2012; Lafortune et al., 2012; Rolls, 2012; Wimmer et al., 2013; Banks et al., 2016; Clawson et al., 2016; Mander et al., 2017). However, neither the origin nor the physiological significance of age-dependent changes in sleep is fully understood, and the question remains whether aging is associated with a decreased capacity to generate sufficient sleep or a reduced sleep need (Mander et al., 2017). Based on this, our study had two overarching aims: (1) to determine whether the age-dependent changes observed at the level of EEG could be explained by the differences at the level of underlying neural activity; and (2) to determine the role of local network mechanisms in the previously observed global sleep disruptions. We predicted that, if the aging process targets neocortical circuits in the first place, this would manifest as a decreased occurrence of network OFF periods during sleep, reduced homeostatic response at the level of local neural activity, or an overall reduced level of neural spiking. To our surprise, we demonstrated that, although aging in mice is consistently associated with global changes in the amount of sleep and its architecture, it does not profoundly affect cortical neural activity. The only changes we observed were increases in the incidence of local LFP slow waves and corresponding population OFF periods during NREM sleep with aging, and that a larger proportion of neurons discharged at a higher rate upon sleep onset. Otherwise, vigilance state-related patterns of cortical activity, and the homeostatic response to SD measured at the level of neuronal population activity during sleep, were intact in older animals. Our results suggest that local cortical mechanisms of sleep regulation are not significantly impaired during healthy aging, making it unlikely that the global sleep disruptions identified with aging arise from changes in local cortical activity. We must therefore critically reconsider the notion that local cortical mechanisms of slow-wave generation are deficient in older humans, at least until intracranial recordings in older human patients can be performed. Furthermore, our results may shed light on why earlier studies report conflicting results, specifically with respect to findings obtained in humans and laboratory animals.

One of the most intriguing observations of our study is that aging has few effects on cortical local neural activity despite profound effects on global brain activity and the daily sleep–wake architecture, as identified in numerous studies (Dijk et al., 1989; Bliwise, 1993; Landolt et al., 1996; Swaab et al., 2002; De Gennaro and Ferrara, 2003; Feinberg and Campbell, 2003; Altena et al., 2010; Colrain et al., 2010; Carrier et al., 2011; Mander et al., 2013, 2014; Martin et al., 2013; Dubé et al., 2015; Clawson et al., 2016), as well as in this study. Evidence suggests that sleep and slow wave oscillations emerge at the level of local neuronal populations as a direct consequence of fine synaptic modifications or local changes in neuronal connectivity arising from specific preceding waking activities (Krueger and Tononi, 2011; Vyazovskiy and Harris, 2013). Therefore, it could be expected that, if this regulatory mechanism is impaired with aging, it should manifest as local disruptions in cortical population activity. However, we found that, in older animals, local neuronal networks are capable of entering and sustaining consolidated OFF periods associated with local slow waves (Fig. 3), and so this basic property of neuronal networks remains intact with aging. Furthermore, we observed an increase in the incidence of slow waves and OFF periods in older animals, which may be indicative of increased sleep pressure (Esser et al., 2007; Riedner et al., 2007; Vyazovskiy et al., 2007), as has been previously suggested based on EEG data (Panagiotou et al., 2017). Although the circuit mechanisms behind the increased tendency of a network to enter OFF periods remains to be determined, we speculate that altered network excitability may play a role (Chen et al., 2012; Lemieux et al., 2015; Neske, 2016). Consistent with this hypothesis, it has been shown that aging is associated with a loss of synaptic connections or a reduction in their stability (Morrison and Baxter, 2012; Grillo et al., 2013). It is possible that the higher incidence of local slow waves we observed in older mice may also have a functional role, such as in the homeostatic rebalancing or remodeling of synaptic networks, which has been associated with sleep slow waves (Chauvette et al., 2012; Krueger et al., 2013; Vyazovskiy and Harris, 2013; Tononi and Cirelli, 2014; Watson et al., 2016; Sanchez-Vives et al., 2017). However, we also observed that the homeostatic rebound of both slow waves and neuronal OFF periods did not manifest marked differences between age groups in our study. This observation is intriguing and contrasts with previous reports of reduced homeostatic rebound in EEG SWA in humans and mice (Wimmer et al., 2013; Mander et al., 2017). It is possible that the direct comparison of changes in local network activity and “global” EEG may not be adequate or precise enough to determine the local mechanistic changes with aging, as age-dependent changes may selectively target specific levels of organization while sparing others. Although we performed recordings of LFP and MUAs from the primary motor cortex, it remains to be established whether associative areas, such as the prefrontal cortex, or primary sensory areas would show similar effects. We speculate that specific cortical and subcortical regions may be differentially affected by aging, with some areas more sensitive than others.

Earlier studies have revealed numerous notable discrepancies between species with respect to the effects of aging on sleep. For example, EEG SWA has been reported to be decreased in older humans (Landolt et al., 1996), whereas recent evidence suggests that in older mice SWA may instead be enhanced (Panagiotou et al., 2017), which was confirmed in our study. On one hand, this may reflect species differences in the effect of aging on sleep need or the capacity to produce deep sleep stages (Klerman and Dijk, 2008). For example, differences between humans and mice may be related to their body and/or brain size and differences in their metabolic rates, which are associated with longevity and sleep (Zepelin and Rechtschaffen, 1974; Allison and Cicchetti, 1976; Siegel, 2005; Capellini et al., 2008; Herculano-Houzel, 2015). Given the major role of ecological factors, such as diet and the risk of predation, on the duration of sleep (Allison and Cicchetti, 1976; Siegel, 2005; Tobler, 2005; Capellini et al., 2008), it cannot be excluded that these factors play an increasingly important role in aging. At the same time, the capacity to engage cortical networks in global sleep may be differentially affected in organisms with different brain sizes, regardless of whether the rate of age-dependent synaptic loss is the same. Based on the evidence in our study, we propose that different species may compensate for the inability to engage in deep consolidated sleep in different ways. For example, older humans may allow intrusions of sleep-like patterns of activity into the awake state, whereas older mice may increase the daily amount of global behavioral sleep. In addition, the spatial scale of the recording methods used to record brain activity in humans and mice differs substantially, with a single scalp electrode in human studies likely recording activity from a larger network than a microwire electrode implanted intracortically in mice. To the best of our knowledge, intracranial recordings from older humans have not been performed during sleep, and so it remains to be determined whether the lack of age differences in neuronal activity can be generalized across species. It is possible that older humans may be perfectly capable of generating fully fledged local slow waves and may indeed have an intact local response to sleep loss.

An important conclusion of our study is that the mechanisms underlying local sleep regulation are distinct from those changes implicated in 24 h sleep–wake control. The effects of aging on the amount and distribution of waking and sleep may be caused by a variety of factors, such as a weaker entrainment to the light-dark cycle or disruptions to the circadian system (Kondratova and Kondratov, 2012; Banks et al., 2015, 2016; Nakamura et al., 2015), fundamental changes in waking behavior (Gu et al., 2015; Fisher et al., 2016) or energy homeostasis (Rolls, 2012). Notably, older mice typically have an increased body weight and reduced locomotor activity (Kopp et al., 2006; Banks et al., 2015), which we also demonstrate here and which may be important in determining the overall sleep–wake architecture. It has recently been shown that providing mice with RWs restores the daily distribution of waking and sleep and improves the capacity to maintain consolidated waking periods (Gu et al., 2015). In our study, all animals were housed with RWs for the duration of the experiment; and although we did not specifically manipulate the amount and speed of running, we noted that the older animals not only ran in the wheels less but they also did not attain the same running speed as younger mice. However, all three age groups consistently showed a negative relationship between the firing rates of individual putative neurons and running speed, as we described previously in younger animals (Fisher et al., 2016). Therefore, we surmise that, because the older animals were less able to sustain consolidated wake periods, possibly as a result of a diminished propensity for running, this resulted in less time spent in the “idling” default wake mode with reduced cortical activity, as we previously suggested (Fisher et al., 2016).

In conclusion, we report that healthy aging in mice does not lead to marked changes in vigilance state-related local neural activity, despite pronounced global changes in the daily amount and distribution of waking and sleep. By and large, most basic features of cortical activity during sleep were not altered by aging, suggesting that powerful protective or compensatory mechanisms may exist to maintain neural function in the neocortex across the lifespan. Crucially, our results suggest that older mice have an intact capacity to generate slow waves and a homeostatic response to sleep loss at the local cortical level, whereas the global sleep dynamics appear to be profoundly disrupted. Therefore, this study importantly highlights that sleep disruption in aging cannot be fully understood without taking into consideration the level of organization and causal relationships or lack thereof between local and global aspects of sleep regulation.
